# Rural-urban differentials in women empowerment and experience of under-five mortality among mothers in Nigeria: a Multiple Indicator Survey analysis

**DOI:** 10.1186/s12889-025-23412-w

**Published:** 2025-07-02

**Authors:** Praise Ooreoluwa Onakalu, Funmilola Folasade Oyinlola, Omolayo Bukola Oluwatope, Ifedapo Agbeja, Immanuel O Shittu, Gbemisola Bolanle Ogbeye, Kwala Adline Okorafor

**Affiliations:** 1 Department of Demography and Social Statistics, Faculty of Social Sciences, Obafemi Awolowo University, Ile-Ife, Osun State Nigeria; 2 The Khana Group, Abuja, Nigeria; 3 Department of Nursing, Faculty of Basic Medical Sciences, Federal University, Oye-Ekiti, Ekiti State Nigeria; 4 Federal Ministry of Health, Abuja, Nigeria; 5 National Centre for Technology Management, Obafemi Awolowo University, Ile-Ife, Nigeria

**Keywords:** Empowerment, Mothers, Under-five mortality, Rural-urban disparities, Social independence, Attitude to violence, Nigeria

## Abstract

**Background:**

Under-five mortality remains a significant concern in low and middle-income countries, with sub-Saharan Africa accounting for 57% of all under-five deaths. Nigeria has one of the highest under-five mortality rates, with approximately 107 deaths per 1000 live births due to preventable causes. Empowering women is a crucial strategy for improving child survival, but there are notable variations in women’s empowerment across Nigeria, which have profound implications for maternal and child health. This study investigated the differences in women’s empowerment between rural and urban areas and its impact on under-five mortality in Nigeria.

**Methods:**

This cross-sectional study utilized secondary data from the 2021 Nigeria Multiple Indicator Cluster Survey (MICS), with a weighted sample size of 38,586 women of reproductive age. Respondents’ characteristics were described by summary statistics, relationships between variables were examined using chi-squares and logistic regression analysis while controlling for potential confounding factors. Analysis was performed using Stata version 17.

**Results:**

The study found that urban residents reported lower under-five mortality rates across most socio-demographic factors. Empowered women, regardless of their residence, were less likely to experience under-five mortality compared to non-empowered women. However, rural women exhibited lower levels of empowerment and social independence compared to their urban counterparts and were more likely to justify domestic violence. Women’s empowerment has a significant influence on the experience of under-five mortality, revealing that women who reported being empowered are 27% less likely (OR = 0.731, *p* = 0.001) to experience under-five mortality as compared to those not empowered.

**Conclusions:**

The study highlighted significant rural-urban disparities in women’s empowerment and under-five mortality rates in Nigeria. To improve under-five mortality rates, interventions focusing on mothers’ social independence and empowerment (socially, economically, and educationally) with a particular emphasis on promoting women’s empowerment in rural areas. Targeted interventions should also address poor societal attitudes towards violence.

## Introduction

The under-five children are among the age groups vulnerable to high rates of mortality, due to their low level of immunity, which predisposes their health to risk of infection and death [1]. Recognising this threat to life, the Sustainable Development Goal 3 (SDG3) by the year 2030, focused on reducing under-five mortality rates to 25 or fewer cases per 1000 live births [2]. Despite the decline that has taken place across the decades, under-five mortality (U5M) remains of global concern as about 4.9 million under-five children died in 2022, by implication, over 13,000 children of this age dying daily [3]. Leading causes of this mortality evolve around prematurity, pneumonia, birth asphyxia, malaria, diarrhea, congenital anomalies injuries, sepsis, tuberculosis, measles, meningitis/encephalitis, HIV/AIDS, tetanus among others [4]. Notably, most of these causes can be preventable through optimal healthcare and affordable, accessible, and cost-effective interventions [5, 6].

Despite global improvements in child survival rates, Sub-Saharan Africa still contributes 57% of the global U5M [4]. Nigeria also has not been excluded from the challenges with approximately 107 deaths per 1000 live births [4]. Even at the regional level, the variation is quite alarming as northern Nigeria has been known for a high rate of U5M compared to the southern region [7]. This figure underscores the need for a deeper understanding of the determinants of under-five mortality, particularly through the lens of women’s empowerment, which has been identified as a crucial factor influencing child survival.

Recently, the National Bureau of Statistics reported that Sokoto State had the highest child mortality rate at 109 per 1,000 live births among weighted children, while Akwa Ibom had the lowest at eight per 1,000 live births. For unweighted children, Oyo State recorded the highest rate at 180 per 1,000 live births, and Lagos State reported the lowest at 0.0 per 1,000 live births. According to the 2021 MICS, the under-five mortality rate was 102 per 1,000 live births. Among weighted children, Lagos State had the lowest under-five mortality rate at 15 per 1,000 live births, whereas Kebbi State had the highest at 179 per 1,000 live births. In terms of unweighted children, Abia State had the highest mortality rate, with 115 deaths per 1,000 live births, while Anambra State had the lowest at 22 per 1,000 live births [8, 9].

Women’s empowerment is a multidimensional concept, encompassing economic, social, political, and cultural areas, which significantly influences different areas of family well-being and child health outcomes inclusive [10]. In a country like Nigeria, with pronounced rural-urban variation, these disparities in empowerment can have profound implications for maternal and child health [11, 12].

Empowerment in this context includes a woman’s ability to make decisions regarding health care, autonomy in household decisions, access to resources, and control over reproductive choices [13]. Studies have shown that empowered women are more likely to seek timely health care services for themselves and their children, engage in health-promoting behaviours, and exercise greater control over family resources, all of which contribute to better child health outcomes [14, 15]. However, significant rural-urban disparities exist in Nigeria, with rural women generally less empowered than their urban counterparts due to limited access to education, healthcare, and economic opportunities [7, 16].

Beyond identified risk factors, U5M is also influenced by various environmental, individual, household, communal, and regional factors [17, 18]. At the community level, rural-urban differentials have been recognized as significant predictors of under-five mortality [19–21]. For instance, a study noted that children in rural areas in Chad have a lower likelihood of under-five mortality compared to their urban counterparts [19], which seems contradictory to the findings of another where rural-dwelling children of Bhutan in South Asia faced higher mortality risks [21]. Additionally [22], highlighted the rural-urban disparity across 35 countries, noting a higher prevalence of under-five mortality in rural areas.

In Nigeria, under-five mortality has been notably higher in rural areas than in urban [7]. These rural-urban differentials have further reflected in the disparities in under-five mortality rates. Urban areas in Nigeria, characterized by better access to health infrastructure and services, have lower under-five mortality rates than rural areas [23, 24]. Moreover, rural women are worse off with additional challenges, such as cultural infiltration of gender norms, lower educational attainment, and restrained decision-making power, which hinder their ability to access and utilize healthcare services for themselves and their children [25].

Household and parental factors, such as housing conditions, also play a pivotal role. Children from homes constructed with inadequate or moderate materials are at higher risk of under-five death compared to those in houses with better-quality materials [26]. Other significant factors associated with under-five mortality include maternal education and age, household size, media exposure (newspaper and television), wealth index, marital status, total children ever born, age at first birth, geopolitical zone, and paternal education and occupation [19–22, 27].

Health-related factors peculiar to maternal conditions have also been traced to U5M, as variables such as delivery by caesarean section, maternal obesity, and the skill level of birth attendants have been shown to influence under-five mortality [27–29]. At the individual level, factors like the child’s sex, birth order, birth size, and birth interval have similarly been linked to under-five mortality outcomes [26, 28, 30, 31] These findings highlight the diverse and contextual nature of under-five mortality and emphasise its role in child health outcomes.

Previous studies have offered valuable insights into the trends, patterns, and predictors of under-five mortality across different countries [3, 19, 32], while also emphasizing its implications for achieving the Sustainable Development Goals [33]. A significant area of interest has been the rural-urban divide in predictors of under-five mortality [21, 22], with some research, such as that by [34] in India, focusing specifically on maternal education. Other studies have explored the role of women’s empowerment in reducing under-five mortality from a global perspective [35, 36] and examined the influence of parental education on child survival [20, 34]. Additionally, the impact of women’s empowerment on broader child health outcomes has been investigated [14, 37].

In the Nigerian context, studies have similarly investigated infant and under-five mortality and its associated factors using datasets across different years and units of the Nigeria Demographic and Health Survey (NDHS) [27, 28, 38]. Some research has focused on specific regions within the country [5] or child survival [39], while others have examined household-related predictors [26] and the impact of women’s empowerment on child health outcomes [11]. Given the breadth of factors influencing under-five mortality, it is essential to analyze the relationship between women’s empowerment and under-five mortality in Nigeria, particularly in the context of rural-urban disparities. However, a dearth of studies explored the influence of women empowerment on under-five mortality from a rural-urban variation using the Multiple indicator cluster survey which stands as the most recent nationally representative dataset in the country. Hence, this study aims to answer the question, does women’s empowerment affect mothers’ under-five mortality experience in rural and urban areas?

## Methodology

### Study design

A secondary analysis of the Nigeria Multiple Indicator Cluster Survey (MICS) 2021 was conducted to assess rural-urban differentials in women’s empowerment and under-five mortality experience. The MICS was implemented by the Global MICS program in collaboration with the National Bureau of Statistics and received technical support from the United Nations International Children’s Emergency Fund [9]. This international initiative aims to facilitate the collection of comparable data across countries on various aspects of women’s and children’s well-being, including child mortality, health, nutrition, education, social protection, maternal health, and empowerment, using a multi-stage sample design. This data is crucial for evidence-based national development strategies, monitoring human development indicators, and alignment with national objectives and global Sustainable Development Goals (SDGs).

## Study setting and participants

The study was conducted in Nigeria. The nation comprises 36 states and the Federal Capital Territory, Abuja, and is characterized by diverse ethnic, linguistic, and cultural groups. Nigeria faces various developmental challenges, including disparities in access to education, healthcare, and social services, particularly among children and women [40]. The study focuses on women of reproductive age, 15–49 years, comprising 28% of the national population. The population is distributed with 51.7% living in rural areas and 48.3% in urban areas, resulting in a population density of 246 people per square kilometer [41–43]. Therefore, it is crucial to include this population in the study to comprehensively understand the impact of under-five mortality experience across rural-urban areas of the country.

## Sampling

The MICS (2021) dataset utilized a multi-stage stratified cluster sampling methodology, employing an updated 2006 census enumeration areas (EAs) as its sampling frame. Primary sampling units (PSUs) were defined as clusters based on these EAs. A systematic sampling approach was applied, selecting 20 households per state, resulting in 1,850 clusters and 37,000 households across the MICS dataset. For this study, a total of 38,585 observations from both rural (20,857) and urban (17,728) areas, derived from the Multiple Indicator Cluster Survey, were used for statistical analysis. The survey provided comprehensive estimates of women’s empowerment and under-five mortality experience among reproductive-age women nationally, regionally, and across rural and urban areas.

## Survey instrument/data collection

This study utilized an adjusted sample size of 38,586 for weighting variables as established in the MICS dataset. Data collection for the 2021 MICS employed a research instrument, encompassing five types of respondents: household; women; men; under-five; and children (aged 5–17). This study utilized the data derived from the women’s questionnaire. Questionnaire content was based on global MICS templates, adapted to the national context by the National Bureau of Statistics in collaboration with relevant government ministries, agencies, departments, and development partners.

## Measures/variables

### Outcome variable

The outcome variable in this study is the experience of under-five mortality which was measured by asking women of reproductive age whether they had a child who later died. This variable was measured dichotomously using Yes [1] and No (0). This response was later recoded as “1” Experienced U5M and “0” Not Experienced U5M [44].

## Independent variable

To validate the extent of association to which the independent variables influence the experience of under-five mortality among women in Nigeria, the independent variables were grouped into socio-demographic factors (such as age, highest level of education, marital status, region, ethnicity, and wealth index) and women empowerment considering social independence and attitude to violence [45, 46]. Age was measured in 5-year age groups as 15–19, 20–24, 25–29, 30–34, 35–39, 40–44 and 45–49. The highest level of education was categorised as “No Education”, “Primary”, “Secondary” and “Tertiary”. Marital status was measured as “Currently in union”, “Formerly in union” and “Never in union”. The region was measured as “North Central”, “North East”, “North West”, “South West”, “South East”, and “South South”. Ethnicity was categorised as “Hausa”, “Igbo”, “Yoruba” and “Others”. Wealth index was classified as “Poor”, “Middle” and “Rich”. The selection of these variables is in line with the literature [38, 47, 48]. The variables reported in MICS 2021 were also considered for the study.

For women’s empowerment, social independence and attitude to violence were considered as used in previous studies [45, 46]. For social independence as one of the women empowerment variables considered for this study: frequency of reading newspapers, frequency of listening to radio, and frequency of watching television were measured as Not at all (0), less than once a week [1], At least once a week [2], and almost every day [3] respectively. In addition, the measure of women’s empowerment considered for this study was the ‘attitude to violence’. This was measured with six questions (such as Going out without telling the spouse, neglecting children, arguing with the husband, refusing sex, burning food, sleeping with another man) which were individually measured dichotomously using Yes as “1” and No as “0”.

Furthermore, a new variable was generated from social independence. The negative option “Not at all” was coded “Low Social independence (0)”, and other options, which ranged from less than once a week [1] to almost every day, were grouped and coded “High Social independence [1]”. Similarly, we constructed another variable from the variable ‘attitude to violence’, where “Yes” to all the questions was categorized as “Positive attitude [1]” and “No” to all questions was categorized as “Negative attitude to violence (0)”. Consequently, a new composite variable was created by merging ‘social independence’ and ‘attitude to violence’ into a single variable, ‘Women’s empowerment’. Negative responses for the two variables were recoded as “Not Empowered (0)”, and positive responses were recoded as “Empowered [1]”.

### Statistical analysis

The study employed Stata version 17 statistical software for data analysis, which involved three phases: univariate, bivariate, and multivariate analysis. The socio-demographic characteristics of the respondents were examined to understand the study population and to organize and check the frequency distribution of respondents, weighted as specified. The dependent categorical variable was presented using bar charts. Bivariate analyses were conducted to explore the association between women’s empowerment and the experience of under-five mortality. The relationship was assessed using chi-square tests of association. At the multivariate level, binary logistic regression was employed, incorporating variables that demonstrated significance (*p* < 0.05) at the bivariate level.

### Handling of the missing values

All missing values (invalid responses) in some variables were acknowledged as missing and, therefore, removed from the analysis.

### Ethical consideration

This study analyzed survey data from UNICEF, where all participants’ personally identifiable information had been removed. The National Statistical Office, the National Bureau of Statistics, and UNICEF obtained informed consent from survey participants before they were involved in the study. In addition, upon completing the registration process, the authors were granted permission to download and use the datasets. The data are available online: https://mics.unicef.org/surveys.

## Results

### Association between socio-demographic characteristics and experience of under-five mortality across rural-urban

Table 1 presents the distribution of under-five mortality experience across key socio-demographic variables, disaggregated by rural and urban residence. Significant differences were observed across all variables. Specifically, maternal age showed a highly significant association with under-five mortality (χ² = 3,730.80; *p* < 0.001), indicating increased experience of child death with advancing age. Educational level was also significantly related to under-five mortality (χ² = 3,186.08; *p* < 0.001), with higher education linked to lower prevalence. Furthermore, marital status demonstrated a strong statistical association (χ² = 3,756.00; *p* < 0.001), suggesting that marital history influences child mortality outcomes. The wealth index revealed significant disparities as well (χ² = 1,394.11; *p* < 0.001). In addition, ethnic background was significantly associated with child mortality (χ² = 1,475.45; *p* < 0.001). Lastly, regional differences in under-five mortality were statistically significant (χ² = 2,189.27; *p* < 0.001), pointing to notable geographic variation in child health outcomes.

**Table 1:** Frequency distribution of Socio-demographic characteristics by experience of Under-Five mortality across Rural-Urban areas

	Rural Area (20,858)		Urban Area (17,728)			Total (38,586)	
	Experience of Under-Five Mortality		Experience of Under-Five Mortality			Experience of Under-Five Mortality	
Variable	Not Experienced (%)	Experienced (%)	Not Experienced (%)	Experienced (%)		Not Experienced (%)	Experienced (%)
**Socio-demographic Factor**							
**Age Group**							
15–19	4,512 (97.69)	107 (2.31)	3,776 (99.66)	13 (0.34)		8,288 (98.58)	119 (1.42)
20–24	3,069 (88.09)	415 (11.91)	2,692 (96.27)	104 (3.73)		5,761 (91.73)	519 (8.27)
25–29	2,582 (77.62)	745 (22.38)	2,396 (91.93)	210 (8.07)		4,978 (83.9)	955 (16.1)
30–34	1,966 (69.06)	881 (30.94)	2,129 (86.95)	320 (13.05)		4,095 (77.33)	1,201 (22.67)
35–39	1,735 (66.05)	892 (33.95)	2,118 (81.95)	467 (18.05)		3,852 (73.94)	1,358 (26.06)
40–44	1,296 (59.93)	867 (40.07)	1,561 (77.09)	464 (22.91)		2,858 (68.23)	1,331 (31.77)
45–49	995 (55.52)	797 (44.48)	1,060 (71.68)	419 (28.32)		2,055 (62.83)	1,216 (37.17)
**Chi-square & p-value**	***χ***^***2***^ ***= 3***,***127.645; p < 0.001***		***χ***^***2***^ ***= 1***,***024.346; p < 0.001***			***χ***^***2***^ ***= 3***,***730.7986; p < 0.001***	
**Level of Education**							
No Education	5,816 (67.12)	2,849 (32.88)	1,093 (68.66)	499 (31.34)		6,909 (67.35)	3,348 (32.64)
Primary	2,495 (72.86)	930 (27.14)	1,411 (76.41)	435 (23.59)		3,906 (74.1)	1,365 (25.9)
Secondary	6,769 (89.4)	802 (10.6)	9,032 (91.68)	820 (8.32)		15,801 (90.69)	1,623 (9.31)
Higher/Tertiary	1,076 (89.88)	121 (10.12)	4,195 (94.56)	242 (5.45)		5,271 (93.56)	363 (6.44)
**Chi-square & p-value**	***χ***^***2***^ ***= 1624.9815; p < 0.001***		***χ***^***2***^ ***= 807.545; p < 0.001***			***χ***^***2***^ ***= 3186.076; p < 0.001***	
**Marital Status**							
Currently in Union	9,869 (69.24)	4,384 (30.76)	7,833 (81.61)		*1*,*765 (18.39)*	17,702 (74.22)	6,149 (25.78)
Formally in Union	723 (71.65)	286 (28.35)	849 (80.48)		206 (19.52)	1,571 (76.16)	492 (23.84)
Never in Union	5,564 (99.42)	32 (0.58)	7,050 (99.64)		26 (0.36)	12,614 (99.54)	58 (0.46)
**Chi-square & p-value**	***χ***^***2***^ ***= 2655.573; p < 0.001***		***χ***^***2***^ ***= 976.039; p < 0.001***			***χ***^***2***^ ***= 3756.001; p < 0.001***	
**Wealth Index**							
Poor	9,644 (73.83)	3,419 (26.17)	750 (78.19)		209 (21.81)	10,394 (74.13)	3,628 (25.87)
Middle	3,560 (80.94)	834 (19.06)	2,541 (81.95)		560 (18.05)	6,101 (81.36)	1,398 (18.64)
Rich	2,952 (86.9)	445 (13.1)	12,441 (91.02)		1,228 (8.98)	15,393 (90.2)	1,673 (9.80)
**Chi-square & p-value**	***χ***^***2***^ ***= 379.295; p < 0.001***		***χ***^***2***^ ***= 223.572; p < 0.001***			***χ***^***2***^ ***= 1394.105; p < 0.001***	
**Ethnic Group**							
Hausa	4,426 (67.29)	2,152 (32.71)	2,600 (79.67)		663 (20.33)	7,026 (71.4)	2,815 (28.6)
Igbo	1,412 (86.34)	223 (13.66)	4,059 (93.42)		289 (6.58)	5,472 (91.49)	509 (8.51)
Yoruba	1,207 (86.64)	186 (13.36)	4,850 (91.43)		455 (8.57)	6,057 (90.43)	641 (9.57)
Others	9,110 (80.97)	2,141 (19.03)	4,223 (87.7)		593 (12.3)	13,332 (82.98)	2,734 (17.02)
**Chi-square & p-value**	***χ***^***2***^ ***= 765.362; p < 0.001***		***χ***^***2***^ ***= 283.722; p < 0.001***			***χ***^***2***^ ***= 1475.446; p < 0.001***	
**Region**							
North Central	3,382 (89.17)	411 (10.83)	1,836 (91.18)		178 (8.82)	5,218 (89.87)	588 (10.13)
North East	3,029 (77.95)	857 (22.05)	1,015 (87.44)		146 (12.56)	4,045 (80.14)	1,003 (19.86)
North West	4,286 (64.3)	2,379 (35.7)	2,369 (77.97)		669 (22.03)	6,654 (68.58)	3,048 (31.42)
South West	1,542 (84.45)	244 (13.55)	5,622 (92.07)		462 (7.59)	7,164 (87.25)	706 (8.97)
South East	1,211 (86.45)	190 (13.55)	2,901 (93.31)		201 (6.49)	4,112 (91.31)	391 (8.69)
South South	2,706 (81.31)	622 (18.69)	1,988 (85.37)		341 (14.63)	4,694 (82.98)	963 (17.02)
**Chi-square & p-value**	***χ***^***2***^ ***= 1429.910; p < 0.001***		***χ***^***2***^ ***= 380.371; p < 0.001***			***χ***^***2***^ ***= 2189.266; p < 0.001***	

Source: MICS dataset, 2021

#### Frequency distribution of women’s empowerment and experience of Under-Five mortality among women of reproductive age

Table 2 explores the relationship between women’s empowerment and under-five mortality among women of reproductive age in rural and urban areas, using indicators such as media exposure, social independence, and attitudes toward partner violence. Overall, higher media exposure measured by frequency of reading newspapers, listening to the radio, and watching television was significantly associated with lower under-five mortality across all populations (p < 0.001). Similarly, greater social independence was linked to reduced child mortality in rural, urban, and total samples. In contrast, justifying wife beating under various scenarios, including going out without informing the husband, neglecting children, arguing, refusing sex, burning food, and infidelity, was consistently associated with higher under-five mortality rates. Notably, rejecting all forms of partner violence emerged as a significant protective factor against child mortality in both rural and urban settings (p < 0.001).

**Table 2:** Frequency distribution of women’s empowerment among women of reproductive age

	Rural (20,858)		Urban (17,728)		Total (38,586)	
	Experience of Under-Five Mortality		Experience of Under-Five Mortality		Experience of Under-Five Mortality	
Women Empowerment	Not Experienced (%)	Experienced (%)	Not Experienced (%)	Experienced (%)	Not Experienced (%)	Experienced (%)
**Social Independence**						
**Frequency of reading Newspaper**						
Not at all	14,922 (76.59)	4,560 (23.41)	11,521 (86.7)	1,768 (13.3)	26,449 (80.69)	6,328 (19.31)
Less than once a week	709 (88.55)	92 (11.45)	2,034 (94.78)	112 (5.22)	2,742 (93.09)	204 (6.91)
At least once a week	458 (90.35)	49 (9.65)	1,615 (94.9)	87 (5.10)	2,073 (93.86)	136 (6.14)
Almost every day	67 (97.46)	2 (2.54)	562 (94.96)	30 (5.04)	629 (95.22)	32 (4.78)
**Chi-square & p-value**	***χ***^***2***^ ***= 161.411; p < 0.001***		***χ***^***2***^ ***= 154.098; p < 0.001***		***χ***^***2***^ ***= 576.168; p < 0.001***	
**Frequency of listening to radio**						
Not at all	10,126 (75.97)	3,203 (24.03)	5,147 (86.94)	773 (13.06)	15,273 (79.34)	3,976 (20.66)
Less than once a week	2,290 (77.99)	646 (22.01)	2,759 (89.07)	339 (10.93)	5,049 (83.68)	985 (16.32)
At least once a week	2,168 (80.76)	516 (19.24)	3,835 (91.14)	373 (8.86)	6,003 (87.1)	889 (12.9)
Almost every day	1,572 (82.36)	337 (17.64)	3,991 (88.63)	512 (11.37)	5,562 (86.76)	849 (13.24)
**Chi-square & p-value**	***χ***^***2***^ ***= 75.929; p < 0.001***		***χ***^***2***^ ***= 30.499; p = 0.004***		***χ***^***2***^ ***= 321.327; p < 0.001***	
**Frequency of watching TV**						
Not at all	10,669 (73.8)	3,787 (26.2)	3,112 (80.66)	746 (19.34)	13,781 (75.25)	4,534 (24.75)
Less than once a week	1,611 (81.97)	354 (18.03)	1,510 (85.52)	256 (14.48)	3,121 (83.65)	610 (16.35)
At least once a week	2,056 (85.21)	357 (14.79)	3,239 (89.93)	363 (10.07)	5,295 (88.04)	720 (11.96)
Almost every day	1,820 (89.94)	204 (10.06)	7,870 (92.57)	632 (7.43)	9,690 (92.06)	836 (7.94)
**Chi-square & p-value**	***χ***^***2***^ ***= 498.132; p < 0.001***		***χ***^***2***^ ***= 278.734; p < 0.001***		***χ***^***2***^ ***= 1***,***471.284; p < 0.001***	
**Social Independence**						
Low social Independence	9,061 (73.63)	3,246 (26.37)	2,154 (80.59)	519 (19.41)	11, 215 (74.87)	3,765 (25.13)
High social Independence	7,094 (82.97)	1,456 (17.03)	13,578 (90.19)	1,477 (9.81)	20,672 (87.57)	2,934 (12.43)
**Chi-square & p-value**	***χ***^***2***^ ***= 316.288; p < 0.001***		***χ***^***2***^ ***= 145.888; p < 0.001***		***χ***^***2***^ ***= 1***,***029.042; p < 0.001***	
**Attitude to Violence**						
If she goes out without telling Husband: wife beating justifies						
No	13,321 (78.94)	3,554 (21.06)	14,676 (89.47)	1,727 (10.53)	27,996 (84.13)	5,281 (15.87)
Yes	2,835 (71.18)	1,148 (28.82)	1,056 (79.65)	270 (20.35)	3,891 (73.3)	1,418 (26.7)
**Chi-square & p-value**	***χ***^***2***^ ***= 139.172; p < 0.001***		***χ***^***2***^ ***= 82.575; p < 0.001***		***χ***^***2***^ ***= 373.916; p < 0.001***	
If she neglects the children: wife beating justified						
No	13,085 (78.78)	3,524 (21.22)	14,469 (89.48)	1,701 (10.52)	27,552 (84.06)	5,225 (15.94)
Yes	3,071 (72.27)	1,179 (27.73)	1,263 (81.06)	295 (18.94)	4,335 (74.63)	1,474 (25.37)
**Chi-square & p-value**	***χ***^***2***^ ***= 103.147; p < 0.001***		***χ***^***2***^ ***= 70.224; p < 0.001***		***χ***^***2***^ ***= 305.357; p < 0.001***	
If she argues with husband: wife beating justified						
No	13,323 (78.76)	3,593 (21.24)	14,534 (89.32)	1,737 (10.68)	27,856 (83.94)	5,331 (16.06)
Yes	2,833 (71.87)	1,109 (28.13)	1,198 (82.23)	259 (17.77)	4,031 (74.66)	1,368 (25.35)
**Chi-square & p-value**	***χ***^***2***^ ***= 109.069; p < 0.001***		***χ***^***2***^ ***= 46.833; p < 0.001***		***χ***^***2***^ ***= 277.846; p < 0.001***	
If she refuses sex with husband: wife beating justified						
No	12,185 (79.84)	3,076 (20.16)	14,027 (89.41)	1,662 (10.59)	26,211 (84.69)	4,738 (15.31)
Yes	3,971 (70.95)	1,626 (29.05)	1,705 (83.59)	335 (16.41)	5,676 (74.33)	1,961 (25.67)
**Chi-square & p-value**	***χ***^***2***^ ***= 232.614; p < 0.001***		***χ***^***2***^ ***= 42.632; p = 0.002***		***χ***^***2***^ ***= 457.908; p < 0.001***	
If she burns the food: wife beating justified						
No	14,508 (78.37)	4,003 (21.63)	15,106 (89.17)	1,834 (10.83)	29,613 (83.53)	5,837 (16.47)
Yes	1,648 (70.22)	699 (29.78)	626 (79.42)	162 (20.58)	2,274 (72.53)	861 (27.47)
**Chi-square & p-value**	***χ***^***2***^ ***= 99.5866; p < 0.001***		***χ***^***2***^ ***= 49.942; p < 0.001***		***χ***^***2***^ ***= 242.755; p < 0.001***	
If she sleeps with another man						
No	7,626 (81.38)	1,745 (18.62)	10,692 (90.97)	1,061 (9.03)	18,317 (86.72)	2,806 (13.28)
Yes	8,529 (74.25)	2,958 (25.75)	5,040 (84.35)	935 (15.65)	13,570 (77.71)	3,893 (22.29)
**Chi-square & p-value**	***χ***^***2***^ ***= 188.432; p < 0.001***		***χ***^***2***^ ***= 121.207; p < 0.001***		***χ***^***2***^ ***= 540.017; p < 0.001***	
**Ave. Attitude to Violence**						
Negative attitude	7,132 (81.77)	1,590 (18.23)	10,224 (91.05)	1,005 (8.95)	17,355 (86.99)	2,595 (13.01)
Positive attitude	9,024 (74.36)	3,112 (25.64)	5,508 (84.74)	992 (15.26)	14,622 (77.98)	4,014 (22.02)
**Chi-square & p-value**	***χ***^***2***^ ***= 200.307; p < 0.001***		***χ***^***2***^ ***= 114.307; p < 0.001***		***χ***^***2***^ ***= 544.856; p < 0.001***	

Source: MICS, dataset, 2021

**Percentage distribution of women’s empowerment and experience of under-five mortality among women of reproductive age across rural-urban areas**.

**Fig. 1:**
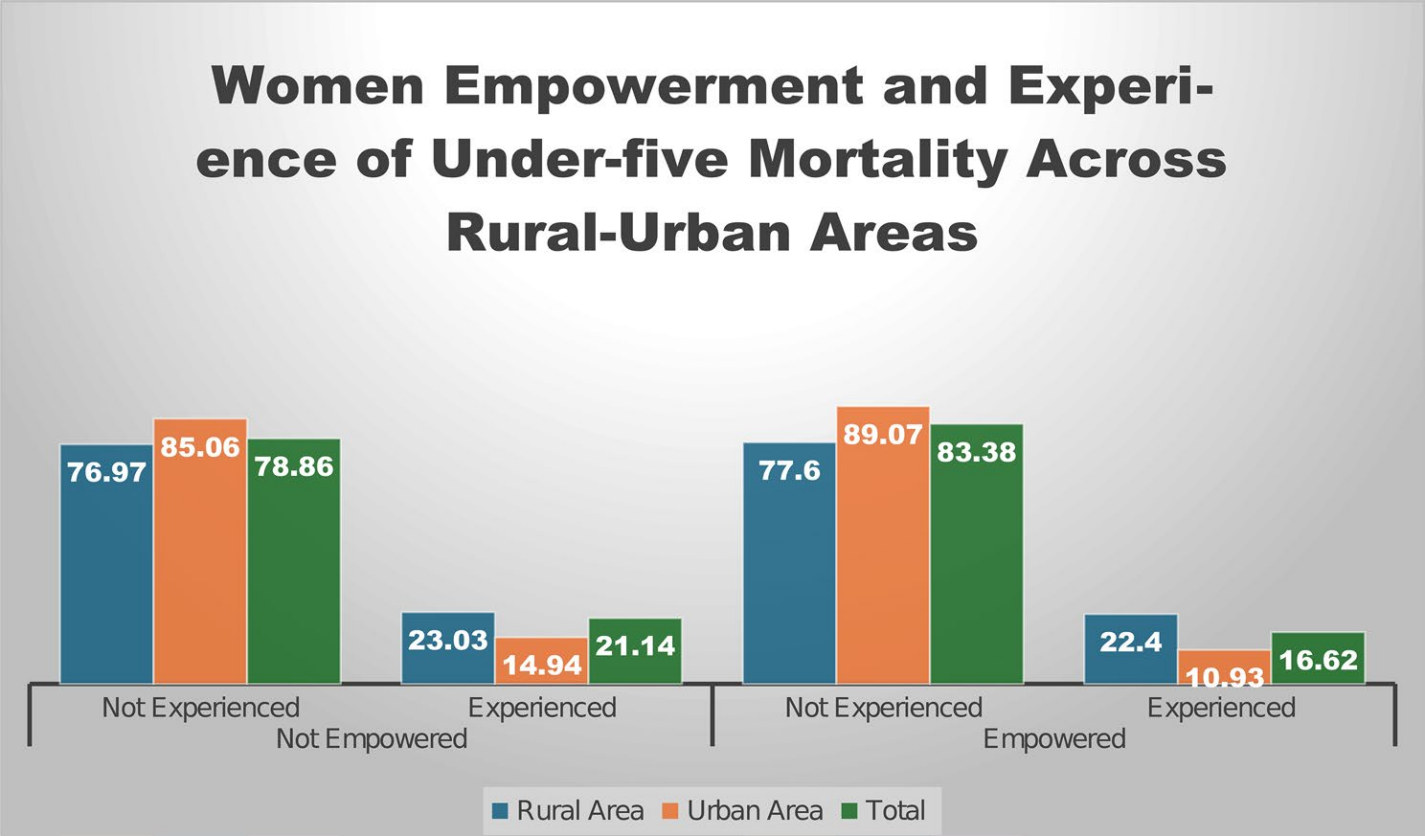
Graphical representation of women’s empowerment and experience of under-five mortality across rural-urban areas

Figure 1 displays a comparative analysis of under-five mortality experiences among women, categorised by empowerment status (empowered vs. non-empowered) and geographic location (rural, urban, and total populations). The data reveal that 76.97% of non-empowered rural women reported no under-five mortality experience, compared to 23.03% who did. In urban areas, these figures improve to 85.06% and 14.94% respectively. Among empowered women, rural areas show 77.6% with no mortality experience versus 22.4% with experience, while urban areas demonstrate better outcomes at 89.07% and 10.93%. The combined totals reflect these trends, with empowered women overall showing lower mortality rates (16.62% experienced) compared to non-empowered women (21.14% experienced).

### Proportion of women who experienced under-five mortality across rural-urban areas

**Fig. 2:**
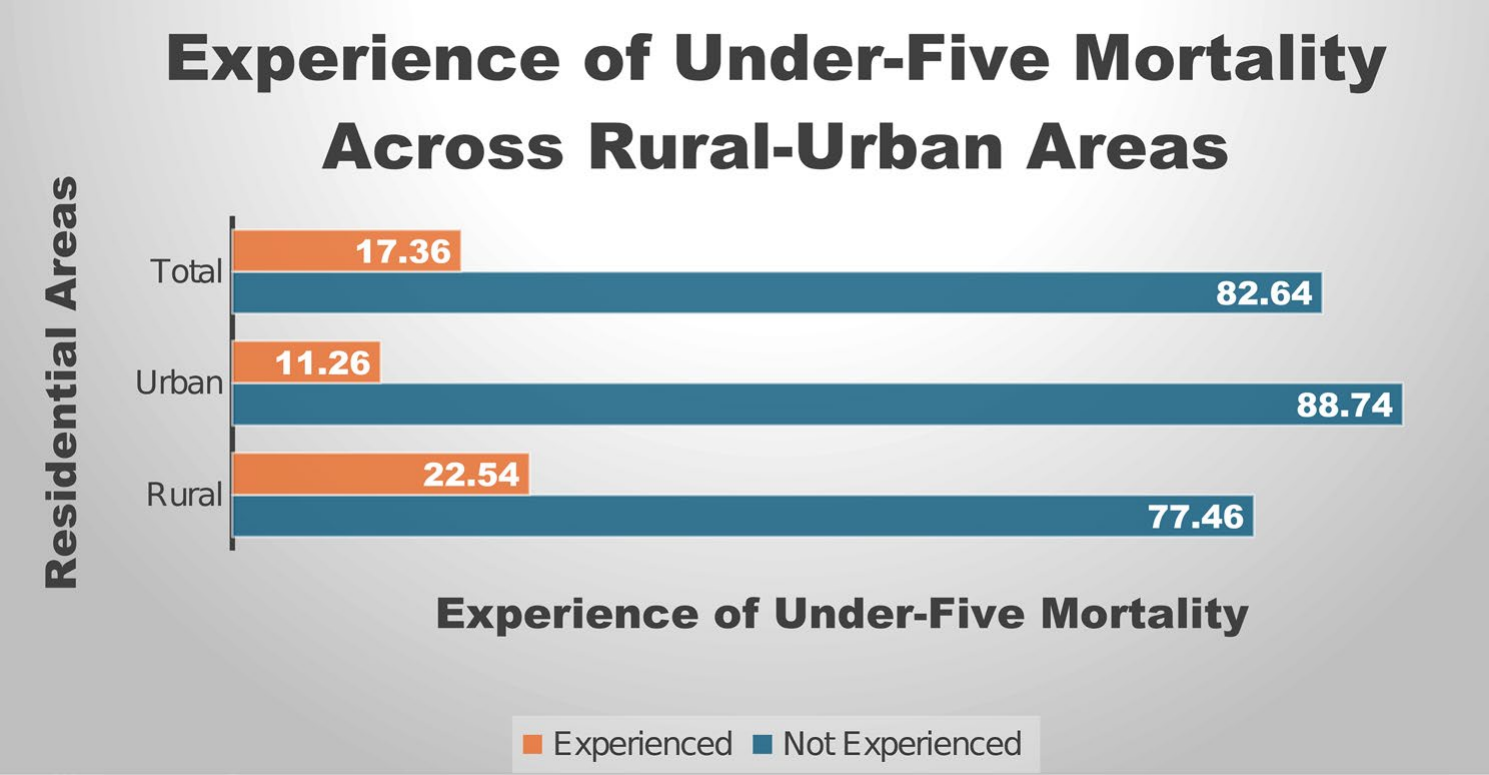
Graphical representation of proportion of experience of under-five mortality among women of reproductive age across rural-urban areas

In measuring the extent of experience of under-five mortality, Fig. 2 revealed the proportion of experience of under-five mortality among women of reproductive age across rural-urban areas of the country. Across rural-urban Nigeria, 22.54% and 11.26% reported to have experienced under-five mortality. In total, 17.36% of the respondents experienced under-five mortality in the survey.

### Multivariate analysis

#### Effects of women’s empowerment on experience of Under-Five mortality across rural-urban areas of Nigeria

Table 3 presents a stratified binary logistic regression analysis examining the relationship between women’s empowerment and the experience of under-five mortality among women of reproductive age in rural and urban areas. Three distinct models were tested to isolate the effects of different empowerment components: social independence (Model 1), attitude toward violence (Model 2), and a composite measure of women’s empowerment (Model 3). The results highlight significant disparities between rural and urban contexts.

In Model 1, which assesses the effects of social independence, women in rural areas with high social independence had 33% lower odds (OR: 0.665, 95% CI: 0.593–0.747) of experiencing under-five mortality compared to those without social independence. The protective effect was even stronger in urban areas, where women with high social independence exhibited 55% lower odds (OR: 0.452, 95% CI: 0.343–0.596). Model 2 evaluates the influence of attitudes toward violence. Women with a positive attitude toward violence faced significantly higher odds of under-five mortality in both rural (OR: 1.919, 95% CI: 1.647–2.238) and urban areas (OR: 1.991, 95% CI: 1.713–2.314). The stronger association in urban areas may reflect heightened risks linked to urban stressors or differing social norms. Also, Model 3 combines these factors into a composite measure of women’s empowerment. Interestingly, while empowerment was associated with a 30% reduction in under-five mortality odds in urban areas (OR: 0.696, 95% CI: 0.583–0.832), it showed no significant effect in rural areas (OR: 1.101, 95% CI: 0.778–1.558).

**Table 3:** Binary logistic regression showing the relationship between women’s empowerment and experience of under-five mortality among women of reproductive age across rural-urban areas

	Model 1 (Effects of Social Independence)				Model 2 (Effects of Attitude to Violence)				Model 3 (Effects of Women’s Empowerment)			
	Rural Area		Urban Area		Rural		Urban		Rural		Urban	
Predictors	OR	95% C. I	OR	95% C. I	OR	95% C. I	OR	95% C. I	OR	95% C. I	OR	95% C. I
**Social Independence**												
Low Social Independence	**(RC)**	**-**	**(RC)**	**-**								
High Social Independence	0.665*	0.593–0.747	0.452*	0.343–0.596								
**Attitude to Violence**												
Negative Attitude					**(RC)**	**-**	**(RC)**	**-**				
Positive Attitude					1.919*	1.647–2.238	1.721*	1.351–2.192				
**Women-Empowerment**												
Not Empowered									**(RC)**	**-**	**(RC)**	**-**
Empowered									0.696*	0.583–0.832	1.101	0.778–1.558

Note: rc = reference category and are in parenthesis; *p < 0.05

## Discussion

This study examined the rural-urban differentials in women’s empowerment and the experiences of women of reproductive age on under-five mortality in Nigeria. A total of 38,585 women from rural and urban areas in Nigeria were included in the study. The findings of the study revealed that women aged 20–24 and 25–29 are more likely to experience under-five mortality. This is consistent with findings from previous studies [38, 49]. This may be attributed to the increase in fertility rates and childcare responsibilities among these age ranges. In contrast, women aged 15–19 reported significantly lower under-five mortality rates, which aligns with the National Demographic and Health Survey (NDHS), 2018 findings. This suggests that younger women may have better access to healthcare services or adopt healthier behaviors [50].

The study’s findings reveal significant associations between socio-demographic variables and under-five mortality in Nigeria, highlighting disparities across rural and urban residences. Maternal age, educational level, marital status, wealth index, ethnic background, and regional differences demonstrate statistically significant relationships with child mortality. These associations underscore the complex interplay of historical, social, cultural, and political factors that shape child health outcomes in Nigeria. From a historical perspective, these disparities reflect legacies of colonialism, uneven development, and unequal distribution of resources, which have created persistent inequalities in access to healthcare, education, and economic opportunities [51]. Socially, cultural norms and practices surrounding marriage, childbearing, and healthcare-seeking behaviors contribute to these disparities, particularly in rural areas where traditional beliefs may influence health practices [52]. For instance, the increased experience of child death with advancing maternal age may reflect the prevalence of early marriage and teenage pregnancies, especially in rural areas, compounded by limited access to family planning and quality maternal care [53]. Politically, regional differences in under-five mortality highlight the impact of governance structures, policy implementation, and resource allocation on child health outcomes, with conflict and insecurity exacerbating these disparities in certain regions [28]. Similarly, the protective effect of higher education on child survival underscores the empowering role of education in improving women’s health literacy and childcare practices [54]. The influence of marital status suggests the importance of social support and economic stability for maternal and child well-being, while disparities linked to wealth and ethnicity point to systemic inequalities in access to essential resources and healthcare [55].

Also, the study showed the complex relationship between women’s empowerment indicators and under-five mortality, specifically examining rural-urban differences. While higher media exposure and social independence correlate with reduced child mortality across both settings, contextual factors significantly shape these associations [22]. In rural areas, limited access to education and healthcare infrastructure may amplify the impact of women’s autonomy and access to information on child health outcomes [28]. Conversely, in urban settings, where resources are relatively more accessible, the influence of these empowerment indicators may be mediated by other factors such as socioeconomic status and environmental conditions [56]. The consistent association between justifying wife beating and increased under-five mortality underscores the pervasive impact of gender-based violence on child well-being, irrespective of geographic location [57]. Critically, these findings call for context-specific interventions that address the unique challenges and opportunities in both rural and urban areas, such as promoting female education and economic empowerment in rural communities, and tackling issues of urban poverty and violence against women in urban centers [58].

This study revealed the disparities in women’s empowerment and under-five mortality outcomes between rural and urban areas in Nigeria. The findings reveal a consistent pattern: women who are empowered are less likely to experience under-five mortality than their non-empowered counterparts, a trend evident across both rural and urban settings. This observation supports existing literature that underscores the critical role of women’s autonomy in improving child health and survival outcomes [59, 60]. Empowered women are generally more likely to access and utilize healthcare services, make informed decisions about child nutrition and immunization, and seek timely medical attention for their children, all of which contribute to better child survival rates [61, 62]. However, the benefits of empowerment appear to be unevenly distributed across geographic regions. In rural areas, the protective effect of women’s empowerment on under-five mortality is relatively muted. This suggests that structural and contextual barriers—such as poverty, poor infrastructure, limited access to quality healthcare, and entrenched gender norms—may constrain the extent to which women can act on their autonomy [63, 64]. In such contexts, even empowered women may find it difficult to translate their decision-making power into meaningful actions that safeguard their children’s health. This limitation underscores the argument that empowerment, while essential, is not a panacea. Without supportive health systems, adequate infrastructure, and community-level gender transformation, the impact of individual empowerment will remain limited [60]. In contrast, urban environments tend to offer a more enabling context for empowered women to exercise their autonomy. Better access to education, healthcare facilities, employment opportunities, and information technologies strengthens women’s capacity to act on their decisions. In such settings, empowerment is more likely to translate into tangible benefits for child health and survival [59]. These findings reinforce the view that the context in which empowerment occurs is as important as the empowerment itself. They highlight the need for place-sensitive strategies that take into account the socio-economic and infrastructural differences between rural and urban areas [64]. Furthermore, the overall lower under-five mortality rates among empowered women affirm the broader developmental importance of women’s empowerment. It is not merely a rights-based goal, but also a practical public health strategy with the potential to reduce child mortality and improve family well-being [61]. Yet, the continued occurrence of child deaths even among empowered women serves as a reminder that empowerment must be accompanied by health system improvements, including access to skilled birth attendants, timely postnatal care, immunization services, and emergency obstetric care [62]. The study also reflects deep-rooted gender inequalities in rural Nigeria, where women often face structural subordination and limited social support. In these settings, empowerment requires more than an individual agency—it necessitates collective action, community engagement, and policy reform to address systemic barriers [63]. Community-based interventions that promote gender equity, improve health literacy, and expand healthcare access are crucial. Additionally, there is a need for targeted policies that prioritize the health and empowerment of rural women, recognizing the compounded disadvantages they face [59, 64].

Further, the study showed the reported extent of under-five mortality among women of reproductive age in Nigeria, as reflected in this study, reveals a critical public health challenge with a clear spatial disparity. The data show a significantly higher experience of child loss in rural areas compared to urban locations. This rural-urban differential is consistent with longstanding evidence that rural Nigerian communities are disproportionately burdened by preventable child deaths [61, 65]. The higher prevalence of under-five mortality in rural areas can be attributed to several structural and systemic factors. Rural communities in Nigeria often suffer from inadequate access to quality maternal and child healthcare services. The distance to health facilities, scarcity of trained personnel, poor transportation infrastructure, and irregular supply of essential drugs and vaccines collectively undermine the survival chances of children in these areas [60]. Moreover, rural households are more likely to experience poverty, malnutrition, and poor water and sanitation conditions, all of which are critical determinants of child mortality. Conversely, the relatively lower levels of under-five mortality reported in urban areas reflect the comparative advantage of urban settings in terms of infrastructure, health education, and access to medical care. Urban women are more likely to deliver in health facilities, complete antenatal visits, and access postnatal care—all of which significantly improve child survival odds [66, 67]. Urban centres also tend to have higher female literacy rates, which enhances health-seeking behavior and awareness of childcare practices. However, the finding that a substantial proportion of women across both rural and urban areas have experienced under-five mortality highlights that this is not an exclusively rural problem. Even in urban settings, issues such as urban slums, overcrowding, and healthcare system inefficiencies persist and contribute to under-five deaths. The persistence of child mortality in these areas suggests that factors beyond mere geographic location—such as household income, maternal education, cultural beliefs, and the functionality of the health system—play an influential role [45, 46]. It is also noteworthy that the overall experience of under-five mortality in the population remains unacceptably high. This calls for urgent multi-sectoral interventions that not only improve access to healthcare but also address the underlying social determinants of health. Strategies should include strengthening the primary healthcare system, enhancing community health worker programs, improving female education, and ensuring the availability of essential maternal and child health services nationwide [68].

The relationship between women’s empowerment and under-five mortality, as revealed through stratified regression analysis at the multivariable analysis level, offers critical insight into the multifaceted nature of health outcomes in Nigeria. The findings clearly demonstrate that components of women’s empowerment—such as social independence and attitudes toward domestic violence—are significant determinants of child survival, but the impact varies considerably across rural and urban contexts. Social independence, as examined in the first model, emerges as a robust protective factor against under-five mortality. Women who exhibit autonomy in decisions related to health, mobility, and information access are significantly less likely to experience the loss of a child under the age of five. This effect is more pronounced in urban areas, likely due to the greater opportunities for empowered women to act on their independence. Urban environments generally provide better access to healthcare facilities, more supportive legal frameworks, and exposure to progressive gender norms that validate women’s decision-making autonomy [69, 70]. In contrast, rural women may face significant limitations that suppress the benefits of autonomy, including physical distance from healthcare services, restricted mobility, and community resistance to female independence [45]. The second model further reveals the deeply rooted challenge of gender-based violence and its implications for child health. A woman’s acceptance of wife-beating—a proxy for internalized gender inequality—is strongly associated with an increased risk of under-five mortality across both rural and urban areas. This finding suggests that women who normalize violence may have lower self-esteem, limited control over reproductive and childcare decisions, and diminished ability to seek healthcare for themselves or their children. In the Nigerian context, where patriarchal structures still dominate, this result underscores the urgency of shifting societal norms that condone or tolerate domestic violence. While such attitudes may stem from longstanding cultural and religious practices, their health consequences are far-reaching and generational [71, 72]. The composite measure of empowerment in the third model offers a more complex understanding. While empowerment significantly reduces the likelihood of under-five mortality in urban areas, it has no significant effect in rural contexts. This discrepancy is particularly telling. In rural Nigeria, empowerment may be more nominal than functional—women might express independence in theory but lack the resources, institutional support, or community validation to act on it effectively. For example, a rural woman may believe in her right to make healthcare decisions but be hindered by financial dependency, cultural expectations, or unavailability of nearby health services. This finding aligns with prior studies that caution against viewing empowerment as a uniform or universally effective tool without considering the local context [73–75]. Moreover, the urban advantage suggests that empowerment is most effective when embedded in systems that allow women to exercise their agency. In cities, women often benefit from higher education, exposure to media, employment opportunities, and civic participation, all of which reinforce their capacity to make impactful decisions for their families. This reinforces the argument that empowerment interventions must go hand-in-hand with structural transformation—particularly in rural Nigeria—if meaningful health outcomes are to be achieved [76, 77]. Ultimately, the divergent patterns observed between rural and urban Nigeria underscore the need for context-specific strategies in promoting women’s empowerment as a pathway to reducing under-five mortality. In rural areas, interventions should go beyond individual-level empowerment and instead address broader systemic constraints—such as poverty, lack of infrastructure, gender-biased cultural practices, and weak health systems. Without such an integrated approach, empowerment alone will have limited capacity to drive change.

### Strength of the study

The study utilized the MICS data set, a nationally representative household data with a stratified cluster sampling technique. The large-scale national survey provides a reliable source of information on health and well-being indicators, including women’s empowerment and child mortality. The MICS dataset is renowned for its rigorous methodology and representative sampling, which gives an enhancement of the study’s generalizability.

The study’s focus on women’s empowerment, specifically social independence and attitudes toward social violence, offers valuable insights into the complex relationships between gender dynamics and health outcomes. By examining the experience of under-five mortality, the study addresses a critical aspect of child health and well-being, contributing to the understanding of factors influencing this significant public health concern.

The study’s methodological prowess is validated through the analysis of secondary data using appropriate statistical methods. Furthermore, the findings are policy-relevant, aligning with Nigeria’s development goals and global health initiatives. Overall, this study adds to the growing body of research on women’s empowerment and health outcomes, particularly in the Nigerian context, providing valuable evidence for policymakers and stakeholders.

### Limitations of the study

This study has some limitations despite the strengths identified. Firstly, the use of secondary data from the Multiple Indicator Cluster Survey (MICS) dataset limits the study’s ability to control for variables and measurement tools. The dataset’s predefined questions and response categories may not fully capture the complexities of women’s empowerment and under-five mortality, potentially leading to measurement biases. Additionally, the cross-sectional design of the MICS dataset restricts the study’s ability to establish causality between women’s empowerment and under-five mortality. Longitudinal studies would provide more robust evidence of the temporal relationships between these variables. The study’s reliance on self-reported data may also introduce social desirability biases, particularly regarding sensitive topics like attitudes toward social violence. Respondents may provide answers deemed socially acceptable rather than their genuine beliefs and current reality, hence potentially influencing the findings of the study. Furthermore, the study’s focus on social independence and attitudes toward social violence as dimensions of women’s empowerment may overlook other critical aspects, such as economic empowerment, education, and access to healthcare. A more comprehensive examination of women’s empowerment would provide a more nuanced understanding of its relationship with under-five mortality. Lastly, the study’s generalizability may be limited to the Nigerian context, and caution should be exercised when applying the findings to other cultural or geographical settings.

## Conclusion

In conclusion, this study underscores the critical role of women’s empowerment in reducing under-five mortality rates in Nigeria. The findings unequivocally demonstrate that socially independent women and those with negative attitudes toward violence are significantly less likely to experience under-five mortality. These results align with the broader literature emphasizing the transformative power of women’s empowerment in improving health outcomes. By empowering women, Nigeria can break the cycle of poverty, inequality, and mortality, fostering a healthier and more prosperous future for its citizens, particularly, the women folk.

The implications of this study extend beyond academic circles and speak directly to policymakers, program managers, and stakeholders involved in the developmental process of the country. To effectively address under-five mortality, interventions must prioritize women’s empowerment, targeting social independence, physical abuse/violence, and attitudes toward violence. This necessitates a multifaceted approach, encompassing education, economic opportunities, healthcare access, and community engagement. By incorporating this comprehensive strategy, Nigeria can accelerate progress toward achieving the Sustainable Development Goals and safeguard the well-being of its most vulnerable citizens. Ultimately, this study serves as a clarion call to action, highlighting the urgent need to empower Nigerian women as a cornerstone of national development. As Nigeria strives to strengthen its healthcare systems, improve child survival rates, and promote gender equality, the findings of this research offer valuable guidance. By harnessing the potential of women’s empowerment, Nigeria can unlock a brighter future for generations to come, characterized by improved health outcomes, reduced mortality rates, and enhanced socioeconomic prospects.

### Recommendations

To combat Nigeria’s persistently high under-five mortality rates, policymakers and stakeholders must prioritize women’s empowerment through evidence-based interventions. This entails investing in scalable programs that foster social independence, economic empowerment, and violence prevention. Integrated initiatives offering education, vocational training, microfinance, and social support services can empower women to make informed decisions about their health and well-being. Additionally, community-based interventions should focus on shifting harmful gender norms and attitudes, promoting a culture of non-violence and respect for women’s rights.

A coordinated approach is crucial to achieving meaningful change. We recommend establishing a National Women’s Empowerment and Child Health Initiative, bringing together government agencies, international partners, and local organizations to implement evidence-based programs, monitor progress, and evaluate impact. Increased funding for women’s empowerment and child health initiatives, coupled with strengthened healthcare systems and enhanced access to quality services, will be critical to success. By adopting this bold, multifaceted strategy, Nigeria can dramatically reduce under-five mortality rates, yielding significant gains in maternal and child health and paving the way for a healthier, more prosperous future.

## Data Availability

Secondary data (women’s dataset) analysed can be accessed online at https://mics.unicef.org/surveys.

## Abbreviations

EA: Enumeration areas
MICS: Multiple Indicator Cluster Survey
PSU: Primary sampling units
SDG: Sustainable Development Goal
NDHS: National Demographic and Health Survey
TV: Television
U5M: Under-five mortality
UNICEF: United Nations Population Funds
